# Event and Comment

**Published:** 1912-07-15

**Authors:** 


### DENTAL REGISTER.

Vol. LXVI. July 15, 1912. No. 7

### EVENT AND COMMENT.

PROFESSIONAL RANKING. In reading the discussion which was provoked by a paper read before the last meeting of the Institute of Dental Pedagogics on a method of teaching histology, one of the participants delivered himself, in the following terms, of an idea that seemed to be troubling him, and one that we often hear mentioned.

He said in substance, "that dental graduates, as a class do not stand on a par in intellectual acquirements with medical graduates, and neither does the dental practitioner stand on a par with the medical practitioner." And he goes on further to say that our colleges must put forth greater efforts to level up the standards. Now whether these statements are true or not will all depend on how we look at the matter. It should be true that medical men are more scholarly, for two reasons: The first is that they must possess a wider knowledge because their field of practice is necessarily greater, they treat the entire body while the dentist confines his work to a part only: and secondly the medical man's work is largely diagnostic and only to a limited extent technical, he must have knowledge of health and be able to detect disease, before he can apply therapeutics. He must be a scientist, a philosopher and an executive of the highest order to meet successfully the demands made upon him. This is the reason why the better medical schools today are pleading for a thorough literary training as a preliminary to a medical education. Two years at least of literary work in a college and four years of study in a medical college are required for graduates in medicine today, and eighty percent of these graduates spend at least one additional year in hospital work before assuming the responsibilities of practice. The modern physician must of necessity be a cultured man as well as a skillful man to cope successfully with the tremendous responsibilities which he is compelled to face. The practice of medicine is an old and well established profession, and because of its importance and deep concern to humanity in general it is respected, and attracts to itself educated men and such as are willing to assume a life work which requires self sacrifice and intellectual consecration.

It is the recognition of these evident facts that called forth the statement we have quoted, Dentistry is as honorable a calling as medicine, or law, or theology, or any other educated vocation; but it is newer and somewhat limited as to its relative importance. It is not fair to compare these callings in any disparaging manner. They are all doing their parts in the worlds work, and each profession is making practical application of all new discoveries in the sciences and arts as rapidly as they are developed and can be assimilated. Dentistry has made rapid and commendable advancement since its origin, probably excelling all other professions, but only because it is young and has been able to draw on all other professional knowledge extensively for its own growth.

The suggestion made that the dental schools must bring their output up to the same standard of intellectual attainment as the medical schools, is ridiculous; all that dental schools can ever be expected to do is to keep up a continual advancement year by year, just as rapidly as the profession is able to assimilate general knowledge and apply it to the science and art of dental practice. Dental schools can not jump at a bound into centuries of history and tradition, neither can they by a mere faculty fiat establish visionary ideals, they must grow in the natural and sequential order. We must not get impatient, nor loose our courage and self respect. We have fought a good fight and kept the faith, and we must press on to our high calling with patience and confidence.

ADVANCING STANDARDS. Scarcely a medical or dental journal comes to our desk which does not in some form contain a complaint as to professional standards or a suggestion that they should be made better. Every department of our work is involved. Our filling materials are not satisfactory, our crown and bridge work is a menace to good health, we are not good business men, and hundreds of other things are at a low ebb and need boosting. All of this may be needed to excite us to greater efforts or to check us in our destructive tendencies, but it gets tiresom and we feel like resenting the constant nagging suggestions that we are not as good as we ought to be, nor as wise as we pretend to be. It is a curious fact that any one can easily establish to his entire satisfaction that the complainers are not usually the doers. In fact the men who are really accomplishing things seldom bother themselves or others with complaints about standards being too low. Is it practicable for State Examining boards and State Laws, or dental colleges and dental Societies, or even the profession collectively or as individuals to make any distinct advancement by merely resolving that certain conditions should be changed and immediately expect a result that is in accordance with their desires Our leaders may tell us what we ought to know and do, but can they force us to do anything that it pleases us not to do. We sometimes wonder if we are not habitually giving ourselves credit for advancement which we should probably never have made had not our patients urged us in the first place to make things better or more comfortable for them. It may be true as has been before suggested that we can never advance farther or faster than our patrons demand. It may save us from making serious blunders if we will stop and consider all these things before acting hastily.

REORGANIZATION OF THE NATIONAL. Judging from the proceeding of the Illinois State Dental Society and an editorial in the June *Items of Interest* things are not progressing as smoothly as was expected in the reorganization plans. To tell the truth we are not pleased with the plan adopted by the committee, but we had hoped that it would be adopted, as it certainly will be some improvement on the present methods of conducting the society. In our opinion what that organization most needs is the united support of the entire profession, that is that portion at least which is organized and represented in local or state societies. This will be necessary to give the National Organization a national constituency, and make it stand for all professional interests instead of the interests of a limited number.

One of the matters that seems to be giving the Committee much trouble is the publication of a journal in connection with the organization, in fact it seems to be one of the chief arguments in favor of reorganization. It seems to us like roofing the house before the foundations are made. If the National ever gets a repectable number of members it will undoubtedly print its own dental journal, but it seems like working at cross purposes to build the society around or upon a dental journal which does not yet exist. It would be a lot more sensible to build up the society on good professional principles first and then take up the journal proposition.

What we need is a national organization which will make practicable a large membership and attendance on its meetings. Dentists can not afford the time or money either to travel three thousand miles—as the Pacific Coast men will do to attend the next meeting in Washington, D. C.,—for what they get out of a meeting of the National as it is now conducted. The attendance at the National for years has been local rather than national, and of recent years it has been necessary for the hosts and entertainment committees to spend large sums of money to induce the people to attend. The dentists of Cleveland last year raised among themselves and their friends seven or eight thousand dollars to entertain the National Convention. This amount would keep a good scientific man at work all the time for a year and this would undoubtedly be more profitable than boat and automobile rides.

We understand there is a proposition to be made at the next meeting to have the *American Medical Association* take over the publication of the journal for the dentists. We do not see how this will make the profession more independent than it is now, when the society's proceedings are printed by a dental supply house journal. Will the dental profession consent to become a section of the *American Medical Association?* This would be a solution of the publication problem but will it increase the membership or influence of the society? We predict not many dentists will consent to such a program. We have made our way thus far as an independent profession and we are not now so hard pressed that we need to give up our heritage.

It seems to us that there is one way that can be tried out without detriment or much hazard, as it is already well started in a few states. That is to organize thoroughly and as completely as may be all the states on the Illinoise plan or something similar, and then unite these organizations to a National Society which shall embrace them all as a delegate body, in which all states shall have representation pro rata to membership. In this way the National body will be truly representative and it can be effectively organized for political and educational service to the profession of the entire country, on a basis of equity and for the highest interests of the society.

